# Clinical Validation of Rapid Gout Detection Method and Kit

**DOI:** 10.3390/mps4040069

**Published:** 2021-09-30

**Authors:** Amith Anugu, Rebecca Monastero, Sahana Pentyala, Vamiq M. Mustahsan, Yanming Cai, Jason Rosenfeld, David E. Komatsu, James Penna, Lawrence Hurst, Srinivas N. Pentyala

**Affiliations:** 1 Stony Brook Medical Center, Department of Anesthesiology, Renaissance School of Medicine, Stony Brook, NY 11794, USA; Amith.Anugu@stonybrook.edu (A.A.); Rebecca.Monastero@stonybrook.edu (R.M.); sahanapen@gmail.com (S.P.); VamiqMohammed.Snu@stonybrook.edu (V.M.M.); Yanming.Cai@stonybrook.edu (Y.C.); jasonrosenfeld68@gmail.com (J.R.); 2 Stony Brook Medical Center, Department of Orthopedics, Renaissance School of Medicine, Stony Brook, NY 11794, USA; david.komatsu@stonybrook.edu (D.E.K.); James.Penna@stonybrook.edu (J.P.); Lawrence.Hurst@stonybrook.edu (L.H.)

**Keywords:** gout, synovial fluid, handheld microscope, uric acid crystals, silver nitrate

## Abstract

Gout is an inflammatory arthritis, which causes intense, acute pain due to the buildup of uric acid crystals in synovial fluid. The gold standard for gout diagnosis consists of synovial fluid analysis by polarized light microscopy, which is costly, time-intensive, and technique-dependent, therefore meriting a more efficient, inexpensive, and accessible method for diagnosis. We previously developed and validated a novel colorimetric gout detection method and device based on the reduction of silver nitrate by uric acid; here, we clinically validated our method and device using arthroscopically obtained synovial fluid samples from gout patients. We successfully identified uric acid crystals in clinical samples via our colorimetric method, visualized uric acid crystals in synovial fluid via handheld microscopy, and determined that silver nitrate stain did not interfere with the microscopic visualization of uric acid crystals necessary for diagnosis. We also developed and validated a method of processing turbid clinical samples for use in our device to prevent the obscuration of uric acid crystals by suspended material. Our method and device will clinically facilitate the immediate colorimetric diagnosis of gout and the subsequent bedside visualization of uric acid crystals in both ideal and turbid synovial fluid samples, allowing for a point-of-care diagnosis of gout.

## 1. Introduction

Gout is an inflammatory arthritis characterized by the buildup of uric acid crystals in synovial fluid, which can cause long-term destruction of joints if severe or recurrent. Ordinarily, uric acid is excreted either through the kidneys or through the gastrointestinal tract, but in patients with high blood levels, uric acid crystallizes with sodium, causing monosodium urate crystals to deposit in joints, tendons, and surrounding tissues [1]. Gout affects approximately 8.3 million people each year, which in the United States presents as a prevalence of between 3–4 percent in the adult population [2,3]. Risk factors for gout include hyperlipidemia, diabetes, heavy alcohol consumption, frequent red meat consumption, and chronic kidney disease, each of which is highly prevalent in the United States population [4]. Gout causes marked levels of pain, swelling, and impaired mobility during a flare and contributes to 0.2% of ED visits per year in the United States [5]. Due to the functional impairment and severe pain associated with a gout flare, expedited diagnosis is critical for quickly and appropriately ameliorating patient symptoms.

On initial presentation of an acutely swollen, painful joint, differential diagnoses include—but are not limited to—gout, pseudo gout, and septic arthritis. Accurate diagnosis is imperative to provide adequate management of the patient’s condition and to prevent recurrence as well as to rule out more serious pathology, such as septic arthritis, which can progress to systemic involvement. As urate crystals in synovial fluid are not visible to the naked eye, polarized light microscopy of arthroscopically obtained synovial fluid is used to diagnose gout [6]. Synovial fluid is obtained from the affected joint and examined under polarized light microscopy to assess for uric acid crystals, calcium pyrophosphate crystals, or cells indicating infection, which would suggest an alternate diagnosis.

The method of gout diagnosis via polarized light microscopy is expensive, as proper microscopy equipment and trained technicians are required for synovial fluid analysis. Although the method is 100% specific for gout in the proper clinical setting, it is only 85% sensitive. One barrier associated with the technique’s sensitivity may be related to technician/physician error in identifying urate crystals, as crystals are only identified correctly 81% of the time when inspected by trained professionals [7]. Additionally, the process is time consuming; by the time the sample is analyzed, a proportion of the urate crystals in the sample may be dissolved or otherwise deteriorated, decreasing the diagnostic capacity of the test [8]. Typically, polarized light microscopy takes approximately two hours if there is an in-house pathology laboratory, but if the sample must be sent out for analysis, processing can take up to two days. A clinical diagnostic rule is occasionally used in situations where synovial fluid has not been drawn in a patient with a presentation suggestive of gout; however, the scoring values associated with the diagnostic rule only assess risk for gout and therefore do not confirm the diagnosis [9].

Various spectroscopic uric acid detectors are on the market which identify elevated serum uric acid levels [10]. However, it is important to note that these detectors do not diagnose gout and merely suggest a patient’s risk of gout. Due to the lack of diagnostic specificity of these methods, it is unwise to use these as bedside diagnostic methods, as they cannot distinguish between gout, pseudo gout, or septic arthritis. Therefore, there is no available bedside method or rapid test for the diagnosis of gout, and a point-of-care gout detection technique is necessary to improve patient care efficiency. Due to the need for a less expensive, less labor-intensive, and more efficient diagnostic method for gout, we developed a point-of-care colorimetric gout detection method, and designed a low-cost device to utilize our method [11]. Silver nitrate is reduced to silver and turns black upon addition of uric acid, which is the colorimetric basis for our rapid uric acid detection method. For the development of a rapid gout detection device with immobile detection matrices, a 4% gelatin mixture was mixed with 20% ammoniacal silver nitrate and layered on glass chambers, and air-dried for 24 h. Uric acid crystals and uric acid crystal suspension (1 mg/200 microliters) in saline solution were added to individual dried well matrices and images were acquired upon reaction. As this method needs validation with clinical samples, this study was performed to evaluate whether our rapid gout detection method can identify uric acid crystals in synovial fluid.

We also sought to ensure that our method would account for the potential turbidity of the samples, as samples of synovial fluid that are obtained in the hospital or clinic can have suspended material, or debris, present within the fluid. Synovial fluid consists of plasma proteins such as cytokines and binding proteins and lubricant macromolecules including hyaluronic acid and PRG4, but erythrocytes, leukocytes, and shed lining cells can also be present in synovial fluid depending on the etiology of the arthropathy [12]. As gout is inflammatory, it causes leukocyte counts to increase in the synovial fluid, typically above 2000 cells per millimeter cubed [13]. Larger pieces of contaminant tissue, or debris, can be inadvertently introduced into the sample during arthrocentesis, and therefore techniques of uric acid crystal microscopy can benefit from removing debris from the sample in order to optimize crystal visualization [14]. Another aim of this work is to test whether the reagents used in our method, e.g., silver nitrate, would interfere with microscopic crystal visualization and imaging and whether handheld microscopes would be effective in capturing these images. There were no prior reports on the investigation of silver nitrate’s impact on the imaging of uric acid crystals for gout diagnosis, or discussion of uric acid crystal imaging at the bedside. This report seeks to investigate these areas as well in order to optimize our point-of-care gout detection method. In order to verify whether our point-of-care method of gout detection can be used in the clinic and whether synovial fluid uric acid crystals can be detected rapidly, we confined this study to investigate designated gout patients’ synovial fluid samples.

## 2. Methods

### 2.1. Development of Bedside Gout Detection Method and Kit

A description of the development of our novel rapid gout detection method and kit [10] is briefly summarized here. Urate crystals of size 5–25 micrometers were prepared and harvested for use in the experiments as per protocols described by McCarty and Faires, and Mandel [15,16]. Uric acid molecules are heterocyclic compounds containing four amino groups, which can reduce ionic Ag in AgNO_3_ to zero valence, giving a distinct change in color. Ammoniacal silver nitrate solutions of varying concentrations were prepared and tested for effectiveness of detecting urate crystals both in aqueous and stained form, as silver nitrate is reduced to elemental silver upon the addition of uric acid. Alizarin red was also tested for effectiveness of distinguishing gout and pseudo gout, as alizarin red will react with calcium pyrophosphate crystals but not with urate crystals. Silver reduction catalysts were also tested for efficacy, including ammoniacal and nondiamine silver stains, to determine which would most quickly facilitate our method of urate crystal detection. Matrix beds of both gelatin and agar were also developed and tested to allow for ease of use via an immobilized detection matrix. Gelatin gave a clear matrix bed even when stored with AgNO3 for weeks, making it the right material for the matrix. Each of these aspects of our experimentation protocols culminated in images acquired via a Promobile microscope (10–200X magnification) attached to an iPhone. Finally, a low-cost method of this device was designed with an attached magnifying lid for use in resource-limited areas.

### 2.2. Clinical Validation of Bedside Gout Detection Method and Kit

***Summary of approach:*** An Institutional Review Board (IRB) approval was obtained for the project “Efficacy and Clinical Application of a Portable Diagnostic Test in Arthrocentesis for Crystals” (SBU-IRB: 1178449-4). This clinical trial was designed as a prospective study with the objective of validating our bedside gout detection method. Clinical samples from 5 patients earlier diagnosed with gout by Stony Brook Orthopedic Practice were obtained via arthrocentesis in either an orthopedic clinic or hospital setting and were tested via both our rapid gout detection method and the standard-of-care of polarized light microscopy. In this study, we attempted to: (a) determine the efficacy of mobile microscopic imaging in detecting uric acid crystals; (b) ascertain whether the silver nitrate stain used for crystal detection would interfere with crystal imaging; and (c) develop a method for analyzing turbid clinical samples. 

***Study population and informed consent:*** Subjects presenting to the Stony Brook Orthopedic Division Clinics or Stony Brook University Hospital Emergency Department were enrolled if deemed to have high clinical suspicion of inflammatory process; differential diagnoses included gout vs. pseudo gout vs. septic arthritis. Inclusion criteria were as follows: (a) patients aged eighteen and over; (b) patients with a diagnosis of gout, pseudo gout, or septic arthritis confirmed by synovial fluid analysis; and (c) patients with site-specific confirmation of gout. Exclusion criteria included: patients aged under eighteen, patients with diagnosis of inflammatory arthritis, patients lacking documented laboratory studies consistent with gout, patients with no history of gout diagnosis, or patients unable to provide informed consent. Informed consent was obtained and documented by members of the study team, which included attending orthopedic surgeons and orthopedic residents. Upon acquisition of consent, consenting subjects’ medical and radiographic imaging was reviewed to collect basic demographic and gout-relevant information. 

***Clinical sample collection and diagnosis:*** We obtained IRB approval to collect samples from designated gout, pseudogout, and septic arthritis patients. In this study, we confined our analysis to synovial samples collected from previously diagnosed gout patients to validate the point-of-care uric acid crystal detection method. Upon aspiration of an affected joint from consenting study subjects earlier diagnosed with gout, half the volume of the clinical samples were marked for standard synovial fluid analysis using polarized light microscopy for standard-of-care diagnosis, and the other half was tested with our rapid gout detection method for comparison. Diagnosis of gout was confirmed to be the visualization of needle-shaped MSU crystals with negative birefringence utilizing a polarizing filter at 400X magnification. Diagnoses were not made based upon the rapid gout detection method prior to its validation. All patients received standard-of-care treatment regardless of participation in the study. No additional joint aspirate was collected in study participants; the excess remaining fluid that would normally be discarded after synovial fluid analysis and diagnosis was instead used to evaluate our rapid diagnostic method. If the rapid gout detection method that we developed does not work, then we will not be able to proceed with further studies. Differential analysis studies for detecting gout (using silver), pseudogout (using alizarin red), and sepsis (using fluorescent dyes) are ongoing with clinical samples and will form the basis for the next report.

***Microscopy images of uric acid crystals stained with silver nitrate:*** To verify whether silver nitrate interaction with uric acid crystals interferes with acquiring microscopy images, pure uric acid crystals stained with ammoniacal silver nitrate were imaged via laboratory-based polarized light microscopy (Accu-Scope 3000 Gout Analysis Microscope, Commack, NY, USA) at 400X magnification. As previously described [10], 20% silver nitrate solution demonstrated maximum staining when compared to other concentrations, and therefore that concentration was used for the silver nitrate stain in our device.

***Rapid gout detection in clinical samples:*** For this study, we procured synovial fluid from designated gout patients who were undergoing treatment at Stony Brook Hospital. A total of 20 ul of synovial fluid was placed on a silver nitrate/gelatin bed matrix and images were acquired with an iPhone 9 (Apple Inc., Cupertino, CA, USA), Plugable Digital Microscope—200X (Plugable, Redmond, WA, USA), and Accu-Scope Gout Microscope—400X. Crystal images were observed with a particular aim to determine whether silver nitrate interfered with the imaging and whether the quality of the images obtained was adequate using the handheld microscope compared to laboratory-based crystal images.

***Turbid sample analysis:*** Often, joint arthrocentesis results in obtaining turbid synovial fluid. For gout diagnosis, a clear synovial fluid sample is ideally desired. To ensure that the rapid gout detection method would be efficacious in evaluating even less-than-ideal samples, 200 microliters of a turbid clinical sample was centrifuged at 100 g in a desktop centrifuge (Fisher Scientific, Pittsburgh, PA, USA) resulting in solid components and liquid components. The liquid component of the sample was separated from the solid using a pipette and the pellet was re-suspended in phosphate-buffered saline (PBS). Interaction of AgNO3 and NaCl (in PBS) results in the formation of a white turbid product which is counteracted with ammonia to form a coordination complex which is much more soluble and can interact with uric acid readily. The PBS–solid solution was then mixed and tested as described above on silver nitrate pre-coated wells, and images were obtained.

## 3. Results

**Figure 1:**
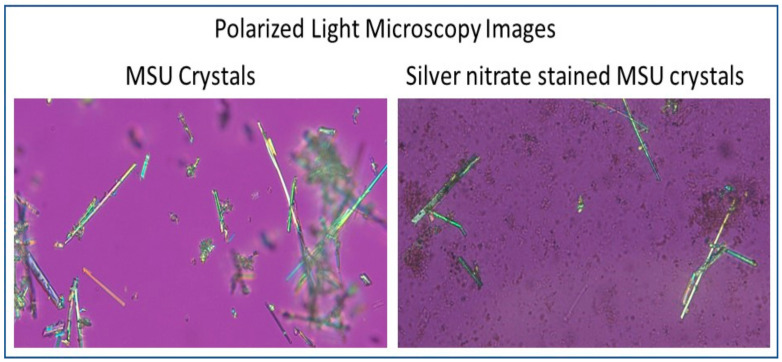
Urate crystals with and without silver nitrate stain under polarized light microscopy.

***Microscopy images of uric acid crystals stained with silver nitrate:*** The reaction of urate crystals with ammoniacal silver nitrate used for staining does not affect the ability to detect and image the crystals under polarized light microscopy at 400X. As demonstrated in Figure 1, the qualities of urate crystals including shape and negative birefringence remain identifiable with silver nitrate stain, allowing for accurate visual diagnosis.

**Figure 2:**
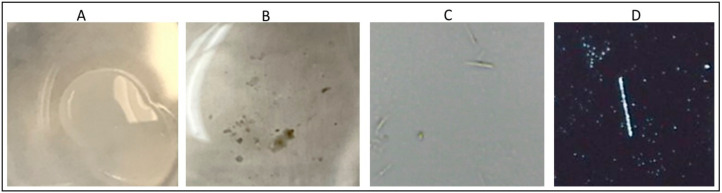
Comparative analysis of clear synovial fluid analysis for detection of urate crystals. Representative images from one sample (n = 5). (**A**) Control; (**B**) iPhone image; (**C**) Plugable Microscope image; (**D**) Accuscope Microscope image.

***Comparative analysis of gout diagnosis:*** Clear synovial fluid samples procured from designated gout patients were imaged after 10 min of placing the samples on the detection matrix with an iPhone (Figure 2B), Plugable Digital Microscope (Figure 2C), and Accu-Scope Gout Microscope (Figure 2D). Within less than 10 min, black spots (Figure 2B) indicating uric acid crystals were observed in the image acquired using an iPhone 9. After 10 min, images that were acquired by handheld plugable digital microscope and Accu-Scope Gout Microscope showed distinct uric acid crystals, distinguished by their interaction with the silver nitrate matrix bed. Figure 2A is an image of the synovial sample on a gelatin bed without silver nitrate (control).

**Figure 3:**
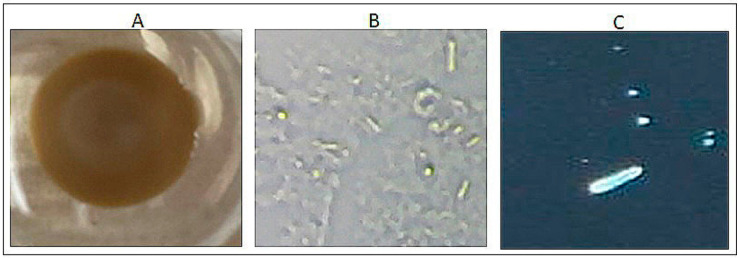
Analysis of turbid synovial fluid for detection of urate crystals. Representative image. (**A**) iPhone image; (**B**) Plugable Microscope image; (**C**) Accuscope Microscope image.

***Turbid sample analysis:*** Turbid clinical samples require processing prior to imaging, due to suspended material interfering with the visualization of uric acid crystals. We standardized a simple centrifugation method (with a desktop centrifuge at 100 g for 5 min at room temperature), and imaged the PBS re-suspended pellet layered onto the silver nitrate matrix bed. Images were obtained after 10 min of placing the turbid sample on the detection matrix with an iPhone (Figure 3A), and of the PBS re-suspended pellet sample with the Plugable Digital Microscope (Figure 3B), and Accu-Scope Gout Microscope (Figure 3C).

## 4. Discussion

Gout is highly prevalent and remains a concern due to the acuity of the pain it causes and the necessity for rapid diagnosis to ensure proper treatment. Risk factors for gout continue to be very common within the population as well, contributing to the continued high prevalence of the disease [17]. The diagnosis of gout, most often accomplished via the gold standard of polarized light microscopy, requires trained technicians, time, and costly equipment. As a result, we recognize the importance of developing and optimizing a point-of-care gout detection method and device, which will improve the cost, time, and effort, associated with the diagnosis or rule-out of gout. Our prior work [11] formed the basis for this technique, and here, we have successfully completed the clinical validation of bedside rapid gout detection. The method we have developed is significant in that it improves upon the gold standard of polarized light microscopy for gout diagnosis; our rapid detection method is an easier, faster, and more accessible way of diagnosing gout, while still retaining the identification of uric acid crystals as the gold-standard foundation for diagnosis. 

Our results demonstrate that ammoniacal silver nitrate staining of uric acid crystals does not interfere with the imaging of crystals. This is in accordance with our hypothesis, as silver nitrate staining by uric acid crystals is dependent upon an oxidation–reduction reaction and does not interfere with crystal morphology or negative birefringence of uric acid crystals, and therefore does not affect their visual identification. These results permit not only the accurate colorimetric diagnosis of gout as previously described, but also permit the clear visualization of the stained uric acid crystals microscopically to facilitate visual diagnosis. In essence, this accomplishes two aspects of gout diagnosis in allowing colorimetric identification of uric acid crystals in addition to the visual identification of crystal morphology, confirming the diagnostic findings in two unique ways. It is also beneficial that our method is effective in crystal visualization, because it indicates that our colorimetric method does not require a trained technician to diagnose gout. Our technique also permits a healthcare provider to visualize the uric acid crystals at the bedside, effectively allowing for any member of the healthcare team to diagnose the patient whether or not they have been trained in polarized light microscopy techniques.

Here, we also demonstrated the important finding of the ability to image crystals using a handheld microscope. We determined that crystal imaging via handheld microscope can be accomplished at 200X magnification and that the morphology and birefringence of uric acid crystals are identifiable at those magnifications to allow for confirmation of diagnosis. The standard of care for gout diagnosis of polarized light microscopy is costly, time intensive, requires trained technicians, and may require samples to be sent to outside laboratories, if the provider’s location does not have the capacity to perform the diagnostic test. Therefore, it is beneficial to demonstrate that our handheld microscope imaging technique is effective in serving this purpose due to its improved cost effectiveness, accessibility, and portability, when compared with standard polarized light microscopy equipment. Both patients and healthcare providers will benefit from faster diagnoses of gout via handheld microscopy of uric acid crystals using our colorimetric method. Patients will be able to receive continued adequate pain relief and the peace of mind given by a quick diagnosis, and providers will be able to facilitate documentation of the images via smartphone for uploading onto an electronic medical record. Further, using this method and the silver nitrate technique/kit with handheld microscopy is beneficial to lower-income areas or underserved communities, because it is less expensive than polarized light microscopic equipment and may provide greater access to care.

Our results have also successfully determined a method for processing turbid clinical samples for use with our diagnostic device. Synovial fluid samples obtained from arthrocentesis are not ideal samples and often contain debris from disrupted tissue due to sampling technique, which can negatively affect the microscopic visualization of uric acid crystals and image quality [14]. In cases where much debris is present, it is possible to incorrectly rule out a diagnosis of gout, if the suspended material eclipses or obscures the examiner’s view of uric acid crystals. Therefore, our successful validation of a centrifugation method of synovial fluid samples has great significance for diagnostic yield. This method, while being extremely effective, is also simple and does not require expensive or advanced laboratory equipment, which further enhances its utility. This aspect of processing the samples remains in line with our goal to use minimal equipment and maximize accessibility and portability while minimizing cost, particularly when compared to polarized light microscopy (approximately USD 3 to 5 for our method vs. USD 40 to 60 for microscopy). The ability to use turbid samples additionally minimizes waste and the need for additional samples to be taken if the first sample is not ideal, which will improve patient satisfaction as well as remove the burden of repeated arthrocentesis by healthcare professionals. 

In this report, we validated the rapid detection of MSU in samples obtained from previously diagnosed gout patients and we expected to see varying amounts of MSU crystals, as was presented in the figures. Uric acid crystals in synovial fluid can be sparse, but the method we developed can identify these crystals from sparse to large amounts, as the interaction with silver changes the uric acid crystals to black, which can be easily visualized. Even if one uric acid crystal is present in the synovial fluid sample, it can be identified with the method that we developed. With this point-of-care gout detection method, appropriate intervention and further testing can be planned by the healthcare provider. Results from ongoing and future clinical trials with a large number of samples will help prove the significance of the rapid MSU detection method for diagnosing gout in patients. It is possible to rapidly detect gout with the method that we developed, but there are a few limitations: (a) Though clear synovial fluid analysis for detecting gout is extremely easy with our method, turbid samples entail additional processing. (b) With our method, the presence of uric acid crystals interacting with silver can be visualized even with the naked eye, but to visualize distinct crystals, devices such as smart phones/tablets with additional optical device attachments are needed. (c) Gout diagnosis can be confirmed by the method that we developed, but if concurrent pseudogout and/or sepsis is to be diagnosed, additional detection methods are needed. Future work will include the additional collection of clinical samples and the use of these clinical samples and simulated synovial fluid in silver nitrate matrix chambers for the determination of the time-based stability and sensitivity of our diagnostic method and device.

Our rapid gout detection method will be compared to the gold standard of polarized light microscopy, and receiver operating characteristic curves will be generated. A high level of sensitivity is expected for our rapid gout detection method due to the colorimetric nature of the test, as only a small sample of synovial fluid is required to identify uric acid crystals. A prospective clinical trial is underway and upon further validation, we anticipate that our novel rapid point-of-care gout detection method will become the standard of care as new smartphones, electronic tablet microscope attachments, and handheld digital microscopes with higher magnification are introduced. This report focuses on the validation of our method to rapidly detect uric acid crystals in clinical samples. Gout diagnosis and eliminating whether the patient has sepsis or pseudogout is the key test characteristic of the method that was developed. Based on rapid gout diagnosis at the bedside, appropriate intervention and further testing can be planned by the healthcare provider. Our ongoing studies using clinical samples to rapidly detect pseudogout and sepsis will lead to the development of a one-step bedside detection method/kit to diagnose all three conditions.
